# Severe Sepsis due to *Clostridium perfringens* Bacteremia of Urinary Origin: A Case Report and Systematic Review

**DOI:** 10.1155/2016/2981729

**Published:** 2016-02-22

**Authors:** Michael A. Millard, Kathleen A. McManus, Brian Wispelwey

**Affiliations:** ^1^Department of Medicine, University of Virginia, P.O. Box 800466, Charlottesville, VA 22908, USA; ^2^Division of Infectious Diseases and International Health, University of Virginia, P.O. Box 801379, Charlottesville, VA 22908, USA

## Abstract

*Clostridium perfringens* bacteremia is an uncommon yet serious clinical syndrome that typically arises from a gastrointestinal source. However, clinicians should consider nongastrointestinal sources as well. We present a rare case of* C. perfringens* bacteremia of urinary origin that required surgical intervention for definitive treatment. A 61-year-old male presented with acute nausea and vomiting, altered mental status, and chronic diarrhea. His physical exam revealed right costovertebral tenderness and his laboratory work-up revealed acute renal failure. Percutaneous blood cultures grew* C. perfringens*. Cross-sectional imaging revealed a right-sided ureteral stone with hydronephrosis, which required nephrostomy placement. On placement of the nephrostomy tube, purulent drainage was identified and Gram stain of the drainage revealed Gram-variable rods. A urinary source of* C. perfringens* was clinically supported. Although it is not a common presentation, nongastrointestinal sources such as a urinary source should be considered in* C. perfringens* bacteremia because failure to recognize a nongastrointestinal source can delay appropriate treatment, which may include surgical intervention.

## 1. Introduction

*Clostridium* species are ubiquitous, spore-forming bacteria that are commonly found in the soil and sewage.* Clostridium perfringens*, the most commonly isolated* Clostridium* species, is an anaerobic Gram-positive rod that is found in the gastrointestinal tract, in the female genital tract, and less frequently on the skin [1]. Clostridia cause a range of infections from food poisoning to soft tissue infections, classically gas gangrene.

*C. perfringens* bacteremia is a rare clinical syndrome, accounting for less than 1% of all bloodstream isolates [2, 3]. Although rare,* C. perfringens* bacteremia carries significant mortality with reported mortality rates ranging from 27 to 58%, and thus it is important to treat it correctly and promptly [2–8].* C. perfringens* bacteremia most commonly arises from a gastrointestinal source [1–4, 9]. Urinary tract infections due to* C. perfringens* have been documented in the literature but are considered rare [3–5, 10–19]. We present a case of an elderly male with* C. perfringens* bacteremia from a urinary source that required surgical intervention for infectious source control.

## 2. Case Presentation

A 61-year-old male with a past medical history notable for diabetes mellitus, chronic kidney disease, hepatitis C, and right below-the-knee amputation due to peripheral vascular disease presented with acute nausea, vomiting, and altered mental status in the setting of chronic diarrhea. His diarrhea had been present for eight months and had been evaluated with a colonoscopy three weeks prior to presentation. The colonoscopy revealed four polyps, all of which were biopsied. Three polyps were tubular adenomas and one polyp in the sigmoid colon was carcinoid.

On admission, physical examination revealed him to be an ill-appearing African American male in moderate distress. He was alert and oriented but unable to recall specific details of his history. His temperature was 37°C, pulse 137 beats per minute, and blood pressure 117/76 mmHg. Examination of the oropharynx revealed dry mucous membranes. Cardiopulmonary exam revealed tachycardia with a regular rhythm and clear lungs to auscultation. He had tenderness to palpation in the right upper quadrant as well as right costovertebral angle tenderness. There was evidence of a right below-the-knee amputation with a well-healed stump.

Laboratory data on admission was notable for a white blood cell count of 15.3 k/uL with 86% neutrophils, hemoglobin of 8.7 g/dL (decreased from 10.4 g/dL six months prior), and platelet count of 64 k/uL (decreased from 253 k/uL six months prior). Haptoglobin was within normal limits making significant intravascular hemolysis unlikely. Serum creatinine was 14.6 mg/dL, which was increased from 3.8 mg/dL six months prior, and blood urea nitrogen was 141 mg/dL. There was an anion gap of 26 with lactic acid of 1.94 mmol/L. Urinalysis revealed 2+ protein, negative nitrites, trace leukocyte esterase, 2 red blood cells/hpf, 3 white blood cells/hpf, no casts, occasional bacteria, and no epithelial cells/hpf.

He was admitted to the medical intensive care unit for severe sepsis and acute renal failure. On admission, percutaneous blood cultures were collected in a sterile fashion and submitted to the microbiology lab in an aerobic bottle and an anaerobic bottle. A BacT/Alert 3D (BioMérieux, Inc., Durham, NC) automated microbial detection system was used to detect bacterial growth. Once growth was detected, the broth was plated on solid media. On hospital day one, two out of four bottles of percutaneous blood cultures grew an organism on anaerobic blood agar that was identified as Gram-positive rods on Gram stain. Vancomycin, ciprofloxacin, and metronidazole were started due to concern for polymicrobial infection. CT abdomen and pelvis without contrast revealed a 5 mm proximal to midureteral stone on the right with associated hydronephrosis, tiny (sub-2 mm) calculi in the bilateral collecting systems, and a small amount of free pelvic fluid along the bilateral paracolic gutters. On hospital day two, Vitek MS (BioMérieux, Inc., Durham, NC), an automated mass spectrometry microbial identification system, identified the Gram-positive organism as* C. perfringens*. Antibiotics were narrowed to ciprofloxacin and metronidazole. On hospital day three, a right percutaneous nephrostomy tube was placed. Placement of the nephrostomy tube returned grossly purulent urine. Gram stain of the right nephrostomy urine revealed Gram-variable rods. Ultimately, no bacteria grew from the right nephrostomy urine culture. Although no bacteria grew on urine culture, the Gram-variable rods are likely* C. perfringens.* The previously administered antibiotics likely disrupted the bacteria's cell wall affecting its Gram staining features.* Clostridium* species may stain as Gram-variable or even as Gram-negative rods as they are decolorized rapidly [20]. Moreover, with right-sided costovertebral tenderness on exam, right-sided nephrolithiasis with hydronephrosis on imaging, and purulent discharge on placement of his percutaneous nephrostomy tube, severe sepsis and bacteremia due to a urinary source are clinically supported. He was treated with ciprofloxacin and metronidazole for fourteen days. Ciprofloxacin and metronidazole were continued, as opposed to narrower coverage with penicillin, because of concern for polymicrobial infection. Surveillance blood cultures remained negative.

## 3. Methods

A PubMed search of the English-language literature was performed in December 2015 using the MeSH term “*Clostridium perfringens*” with each of the following MeSH terms: “bacteremia,” “cystitis,” and “pyelonephritis.” The systematic review revealed 15 cases of urinary tract infections due to* C. perfringens*. There were four cases that did not contain sufficient detail to include in the review. The clinical characteristics including age, gender, comorbidities, presence of bacteremia, antimicrobials used, and outcomes are summarized in Table 1.

## 4. Discussion

*Clostridium* species, including* C. perfringens*, are uncommonly isolated from blood cultures, which can make clinical interpretation difficult. Ingram and Cooper reviewed blood culture data from a large community teaching hospital over a ten-year period and found that Clostridia represented less than 1% of the positive blood cultures [2]. Similarly, Rechner et al. reviewed 63,296 blood cultures from a rural hospital of which 74 (0.12%) were positive for* Clostridium* species [3].

Despite being infrequently encountered,* C. perfringens* bacteremia carries significant mortality and thus remains clinically relevant. The mortality of* Clostridium* bacteremia ranges from 27 to 58% in published retrospective case series [2–8]. Outcomes have been shown to improve with prompt initiation of antibiotics and appropriate antibiotic selection [8].

The patient described in our case was diabetic, elderly, recently diagnosed with a malignancy (carcinoid tumor), and presented with renal insufficiency, all of which are characteristics associated with* Clostridium* bacteremia in published case series. Different case series of patients with* Clostridium* bacteremia report underlying malignancy in 41.9–52% of patients, age over 65 years in 67.4–83.3% of patients, and diabetes in 20–40% of patients [2–9]. Yang et al. reviewed 93 cases of* Clostridium* bacteremia and found that 49.5% of patients had renal insufficiency [4].

Gorbach and Thadepalli performed one of the earliest analyses on* Clostridium* in human infections and found intra-abdominal sources were the most common source of infection (28 of 65 cases) [1]. Several more recent retrospective analyses have reported a bowel or intra-abdominal source in 45–65% of cases. Other less common sources include female genital tract, respiratory tract, and skin/soft tissue [2–4, 9].

*C. perfringens* is an uncommon urine culture isolate that rarely leads to a clinically significant urinary infection. Headington and Beyerlein reviewed 15,250 consecutive clean midstream or catheter urine specimens for the presence of anaerobic bacteria. Anaerobes were isolated in 1% of urine samples.* Clostridium* species were the second most commonly isolated organism representing 29.3% of the isolated anaerobes. Of the* Clostridium* species,* C. perfringens* was the most common bacteria accounting for 89% of the Clostridia isolates. However, the authors report that, in their case series, no infections could be attributed to the positive* Clostridium* urine cultures [21].

Medical literature contains a paucity of clinically significant* C. perfringens* urinary tract infections (summarized in Table 1). In addition, Gorbach and Thadepalli's case series notes one case of a polymicrobial prostatic abscess in which* Clostridium* was isolated [1]. Three other case series note single cases of* C. perfringens* urinary tract infections but do not describe the cases in detail [3–5]. Of the eleven cases with sufficient detail to be included in the systematic review, seven of the eleven (64%) patients were older than 65 years, five out of the eleven (45%) patients had diabetes, two out of the eleven (18%) patients had a malignancy, seven of the eleven (64%) cases had associated bacteremia, and three of the eleven (27%) patients died (Table 1). Compared to published case series of* C. perfringens* bacteremia from all sources including nonurinary sources, the systematic review revealed similar rates of elderly patients and patients with diabetes but a lower percentage of patients with a malignancy. The patient in our case was similar to the patients in the systematic review in that he was elderly and diabetic.

## 5. Conclusion

We present a rare case of severe sepsis due to* C. perfringens* bacteremia of urinary origin. Urine cultures with Gram-positive rods are fairly uncommon and, as a result, may be dismissed as clinically irrelevant. Our case illustrates that* Clostridium* bacteremia can be due to urinary tract infections. In our case, the patient required surgical intervention with placement of a percutaneous nephrostomy tube for source control. Given that* Clostridium* bacteremia and sepsis can have significant mortality, prompt diagnosis, search for a source, including considering a urinary source, and appropriate treatment and interventions are essential in achieving a good outcome.

## Figures and Tables

**Table 1:** Systematic review of urinary tract infections due to *Clostridium perfringens*.

Case	Age/sex	Diabetes	Renal insufficiency	Malignancy	Condition	Bacteremia	Initial antibiotics	Final antibiotics	Outcome
Nielsen and Laursen, 1972 [10]	75/F	Yes	No	No	Cystitis	No	TMP/SMX	TMP/SMX	Survived and infection resolved
Shah et al., 1973 [11]	84/F	No	Not reported	No	Emphysematous cystitis	No	Nitrofurantoin	Penicillin G	Survived and infection resolved
Wayland and Kiviat, 1974 [12]	80/M	Yes	No	No	Emphysematous cystitis	Yes	Not reported	Not reported	Deceased
Wayland and Kiviat, 1974 [12]	62/F	Yes	Not reported	No	Emphysematous cystitis	Yes	Penicillin	Cephalosporin	Survived and infection resolved
Maliwan, 1979 [13]	38/F	No	Not reported	No	Emphysematous cystitis	Yes	Penicillin G	Penicillin G	Survived and infection resolved
West et al., 1981 [14]	72/F	Yes	Not reported	No	Emphysematous cystitis	No	Penicillin G	Penicillin G	Survived and infection resolved
Galloway, 1984 [15]	58/F	No	Yes	Yes	Emphysematous cystitis	Yes	Penicillin	Penicillin	Deceased
Bergman and Warren, 1988 [16]	76/M	No	Yes	No	Cystitis	No	Penicillin, metronidazole, amikacin	Penicillin	Survived and infection resolved
Greene, 1992 [17]	70/M	No	Not reported	Yes	Emphysematous cystitis	Yes	Metronidazole, cefotaxime	Penicillin, cefotaxime	Survived and infection resolved
Katz et al., 1993 [18]	75/F	Yes	Yes	No	Emphysematous cystitis	Yes	Ampicillin	Penicillin G	Survived and infection resolved
Lazarescu et al., 2012 [19]	34/F	No	Not reported	No	Gangrenous cystitis	Yes	Not reported	Not reported	Deceased

## References

[1] Gorbach S. L., Thadepalli H. (1975). Isolation of *Clostridium* in human infections: evaluation of 114 cases. *Journal of Infectious Diseases*.

[2] Ingram C. W., Cooper J. N. (1989). Clostridial bloodstream infections. *Southern Medical Journal*.

[3] Rechner P. M., Agger W. A., Mruz K., Cogbill T. H. (2001). Clinical features of clostridial bacteremia: a review from a rural area. *Clinical Infectious Diseases*.

[4] Yang C.-C., Hsu P.-C., Chang H.-J., Cheng C.-W., Lee M.-H. (2013). Clinical significance and outcomes of *Clostridium perfringens* bacteremia—a 10-year experience at a tertiary care hospital. *International Journal of Infectious Diseases*.

[5] Fujita H., Nishimura S., Kurosawa S., Akiya I., Nakamura-Uchiyama F., Ohnishi K. (2010). Clinical and epidemiological features of clostridium perfringens bacteremia: a review of 18 cases over 8 year-period in a tertiary care center in metropolitan Tokyo area in Japan. *Internal Medicine*.

[6] Leal J., Gregson D. B., Ross T., Church D. L., Laupland K. B. (2008). Epidemiology of *Clostridium* species bacteremia in Calgary, Canada, 2000–2006. *Journal of Infection*.

[7] Pietrafitta J. J., Deckers P. J. (1982). Significance of clostridial bacteremia. *The American Journal of Surgery*.

[8] Shah M., Bishburg E., Baran D. A., Chan T. (2009). Epidemiology and outcomes of clostridial bacteremia at a tertiary-care institution. *TheScientificWorldJOURNAL*.

[9] van Bunderen C. C., Bomers M. K., Wesdorp E., Peerbooms P., Veenstra J. (2010). *Clostridium perfringens* septicaemia with massive intravascular haemolysis: a case report and review of the literature. *Netherlands Journal of Medicine*.

[10] Nielsen M. L., Laursen H. (1972). Clostridial infection in the urinary tract: report of a case of bladder infection due to *Clostridium welchii*. *Scandinavian Journal of Urology and Nephrology*.

[11] Shah M. S., Nabong R., Rogin A. (1973). Sequestration of the total bladder mucosa caused by clostridial infection. *Journal of Urology*.

[12] Wayland J. S., Kiviat M. D. (1974). Clostridial cystitis emphysematosa. *Urology*.

[13] Maliwan N. (1979). Emphysematous cystitis associated with *Clostridum perfringens* bacteremia. *Journal of Urology*.

[14] West T. E., Holley H. P., Lauer A. D. (1981). Emphysematous cystitis due to *Clostridium perfringens*. *The Journal of the American Medical Association*.

[15] Galloway N. T. M. (1984). Gas gangrene of the bladder complicating cyclophosphamide cystitis. *British Journal of Urology*.

[16] Bergman S., Warren N. (1988). Clostridial cystitis in a nondiabetic man: case report. *Virginia Medical*.

[17] Greene M. H. (1992). Emphysematous cystitis due to *Clostridium perfringens* and *Candida albican*s in two patients with hematologic malignant conditions. *Cancer*.

[18] Katz D. S., Aksoy E., Cunha B. A. (1993). Clostridium perfringens emphysematous cystitis. *Urology*.

[19] Lazarescu C., Kimmoun A., Blatt A., Bastien C., Levy B. (2012). Clostridium perfringens gangrenous cystitis with septic shock and bone marrow necrosis. *Intensive Care Medicine*.

[20] Stone R. B., Steele J. C. H. (2009). Impact of reporting gram stain results from blood cultures on the selection of antimicrobial agents. *American Journal of Clinical Pathology*.

[21] Headington J. T., Beyerlein B. (1966). Anaerobic bacteria in routine urine culture. *Journal of Clinical Pathology*.

